# Reassembly of JIP1 Scaffold Complex in JNK MAP Kinase Pathway Using Heterologous Protein Interactions

**DOI:** 10.1371/journal.pone.0096797

**Published:** 2014-05-09

**Authors:** Jiyoung Moon, Sang-Hyun Park

**Affiliations:** Department of Biological Sciences, Seoul National University, Seoul, Korea; Hungarian Academy of Sciences, Hungary

## Abstract

Formation of signaling protein complexes is crucial for proper signal transduction. Scaffold proteins in MAP kinase pathways are thought to facilitate complex assembly, thereby promoting efficient and specific signaling. To elucidate the assembly mechanism of scaffold complexes in mammals, we attempted to rationally rewire JIP1-dependent JNK MAP kinase pathway via alternative assembly of JIP1 complex. When JIP1-JNK docking interaction in the complex was replaced with heterologous protein interaction domains, such as PDZ domains and JNK-binding domains, a functional scaffold complex was reconstituted, and JNK signaling was rescued. Reassembly of JIP1 complex using heterologous protein interactions was sufficient for restoring of JNK MAP kinase pathway to induce signaling responses, including JNK activation and cell death. These results suggest a simple yet modular mechanism for JIP1 scaffold assembly in mammals.

## Introduction

Assembly of protein complexes is thought to facilitate the specificity and efficiency of cell signaling by increasing the proximity among participating signaling proteins [1], [2]. Scaffold proteins in mitogen-activated protein (MAP) kinase pathways play a key role in various cellular responses via complex formation with component kinases. Signaling specificity is precisely controlled in MAP kinase signaling networks, despite the fact that several pathway members are shared among multiple MAP kinase cascades. Physical insulation of active signaling proteins through complex formation mediated by pathway-specific scaffold proteins is postulated to dictate the signaling specificity [3]–[6]. However, the mechanisms of scaffold assembly and its role in generating cellular responses remain unclear. A yeast scaffold protein, Ste5, involved in the mating MAP kinase pathway is known to function as a modular organizing center for the assembly of mating signaling proteins [7], [8]. Previous studies have revealed that individual docking interactions of Ste5 scaffold with its member kinases in the upper tier of the pathway -Ste11 (MAPKKK) and Ste7 (MAPKK)- could be functionally replaced by heterologous protein interactions and that such wiring was sufficient to restore pathway connectivity [9]. However, the docking of Ste5 with the terminal MAP kinase, Fus3, was not replaceable. This finding indicated that the assembly of Ste5 scaffold with MAPKKK and MAPKK of the pathway is mainly controlled by non-covalent interactions, and precise stereophysical arrangements may not be critical for functional assembly of Ste5 complex.

JNK-interacting protein 1 (JIP1) is a canonical scaffold protein in mammalian cells and functions in the JNK MAP kinase pathway, which is composed of MLK3 (MAPKKK), MKK7 (MAPKK) and JNK (MAPK) [10]–[13]. JIP1 scaffold is known to tether all three member kinases in the JNK signaling pathway but shows little sequence similarity with Ste5 scaffold in yeast. JIP1 scaffold contains the JNK-binding domain (JBD), which is conserved in several JNK-interacting proteins, such as MKK4, glucocorticoid receptor (GR), MKK7, and cJun [13]–[15]. Recently, scaffolds in mammalian MAP kinase signaling pathways have been implicated as potential drug targets due to the critical role of scaffold-dependent assembly in signaling [3], [16]. Several studies suggested that docking interactions in yeast scaffold complexes are highly modular [17]–[19]. Nonetheless, assembly mechanisms of mammalian scaffolds are yet to be fully understood.

In this study, to investigate the functional mechanism of the JIP1 scaffold assembly, we have attempted to restore the JIP1-dependent JNK MAP kinase pathway by replacing the JIP1-JNK docking interaction with heterologous protein interaction modules based on PDZ domains or JBDs [9], [20]. We utilized a JIP1 mutant defective in the recruitment of JNK, which led to decreased signaling responses. We demonstrated that the JNK signaling response could be restored when the defective JIP1 assembly was wired by reassembling a functional JIP1 complex using heterologous protein interactions. This finding suggests that JIP1 assembly is highly modular and that the function of JIP1 scaffold is presumably to enforce pathway members into close proximity for the efficient catalysis and insulation of active kinases against unnecessary crosstalks, thereby ensuring specific signaling.

## Materials and Methods

### Cell lines and transfection

293T and COS7 cells were provided by Y.K. Jung [21]. JIP1−/− MEF cells [22] were provided by P.L. Han and were immortalized by transfection with pSV40. 293T, JIP1−/− MEF, and COS7 cells were cultured in Dulbecco Modified Eagle Medium (Gibco) supplemented with 10% (v/v) FBS (Gibco). 293T and COS7 cells were transfected with the appropriate plasmids using Lipofectamine (Invitrogen). Plasmid DNA (0.5–2 µg) mixed with Lipofectamine in DMEM (200 µL) was incubated for 15 minutes and then added to FBS-starved cells. After 2 hours of incubation, the medium was supplemented with DMEM containing FBS. Immortalized JIP1−/− MEF cells were transfected by electroporation using the Neon transfection system (Invitrogen).

### Plasmids for protein expression

The plasmid constructs used for protein expression are listed in Table S7 in File S1. JIP1 gene was cloned from a mouse brain cDNA library using PCR. The sequence of cloned JIP1 gene was confirmed by DNA sequencing and compared to the published sequence (GenBank database accession no. NM_011162). JIP1* and JIP1ΔJBD were generated by introducing point mutations (R156G and P157G) and deleting the JBD (156–164), respectively, in JIP1 by PCR mutagenesis. Myc-tagged JIP1, JIP1* and JIP1ΔJBD were cloned into pcDNA3.1 plasmid for expression. JBDs from JIP1, MKK4 and GR were fused to the N-termini of JIP1 variants using synthetic oligonucleotides with a linker composed of five Gly and Ser residues (Ser-Gly-Ser-Gly-Ser). GFP-tagged JIP1, JIP1*, syn-JIP1* and JBD^JIP1^-JIP1* were also cloned into pcDNA3.1 plasmid. DsRed-tagged JNK1 and JNK1-nNOS were cloned into p3xFLAG-CMV plasmid. Genes encoding the mouse JNK and GST-cJun (1–79) were provided by E.J. Choi, and the mouse MLK3 gene was provided by Y.K. Jung.

### Syn-nNOS PDZ recruitment system

PDZ domains from syntrophin (syn) and neuronal nitric oxide synthase (nNOS) are heterodimeric interaction domains which are well-characterized in previous studies [9], [20]. These interaction modules have a modest binding affinity (dissociation constant *K*_d_ = 0.6 µM) and are not involved in JIP1-dependent JNK signaling pathway. In order to recruit JNK into JIP1*, syn PDZ domain (residues 77–171) was fused to the N-termini of the JIP1 variants with a linker composed of five Gly and Ser residues (Ser-Gly-Ser-Gly-Ser). The corresponding partner fusions, JNK1-nNOS and JNK2-nNOS, were constructed by fusion of nNOS PDZ domain (residues 1–130) with the same linker to the C-terminus of Flag-JNK.

### Immunoblotting and immunoprecipitation

After transfection for 24 hours, cells were lysed in a lysis buffer (50 mM HEPES pH 7.4, 150 mM NaCl, 2 mM EDTA, 25 mM β-glycerophosphate, 1 mM sodium orthovanadate, 1 mM PMSF, 10 µg/mL leupeptin, 1% (v/v) glycerol and 0.1% (v/v) NP-40). Proteins in the samples were separated on a 10% SDS-PAGE and transferred to a nitrocellulose transfer membrane. The blots were blocked in 5% (w/v) skim milk for 1 hour and incubated with primary antibodies in TBST buffer overnight at 4°C. The antibodies used in this study were as follows: anti-phospho JNK (Cell Signaling), anti-Flag (Sigma), anti-HA (Boehringer Mannheim), and anti-myc (Upstate). Proteins were detected with the LAS-3000 (Fuji) imaging system using West Pico Chemiluminescent Substrate (Thermo Scientific) according to the manufacturer's instructions. Myc-tagged scaffold proteins were immunoprecipitated by incubation with an anti-myc antibody conjugated to agarose (Sigma) for 4 hours at 4°C. The samples were washed three times with lysis buffer and examined by western blot analysis. All the western blot analyses were performed at least three times.

### Kinase assay

Protein kinase activity was quantified using an in-gel kinase assay [23]. Samples were separated by SDS-PAGE containing GST-cJun (0.25 mg/mL). The gels were washed with a buffer (20% (v/v) 2-propanol and 50 mM HEPES pH 7.6) and washed again with buffer A (50 mM HEPES pH 7.6 and 5 mM β-mercaptoethanol). Proteins were refolded by incubating gels in buffer A containing decreasing amounts of urea (6–0.75 M) at 25°C for 5 hours, followed by five washes with buffer A at 4°C for 1 hour. Gels containing the refolded proteins were incubated for 15 minutes in a kinase assay buffer (25 mM HEPES pH 7.0, 20 mM MgCl_2_, 20 mM β-glycerophosphate, 0.1 mM sodium orthovanadate, 2 mM DTT, 20 µM ATP and 5 µCi of [γ-^32^P] ATP). After washing and drying, the [^32^P] phosphorylated GST-cJun in gels was visualized using a phosphorimager, and incorporated radioactivity was quantified using a liquid scintillation counter.

### Immunocytochemistry

Cells were fixed using paraformaldehyde after 24 hours of transfection. Flag-tagged proteins were detected by incubation with an anti-Flag antibody for 2 hours, followed by incubation with a TRITC-conjugated anti-mouse IgG antibody (Molecular Probes) for 2 hours. Nuclei were detected by staining with DAPI. Fluorescence images of ∼100 cells were obtained using multi-photon confocal laser scanning microscope (DE/LSM510 NLO, Carl Zeiss) and were processed using LSM Image Browser program (Carl Zeiss), and the representative images were shown in the figures. The localization of JNK1 and JNK1-nNOS in the Figure S4 in File S1 was examined from fluorescence images obtained using DeltaVision system (Applied Precision).

### Cell death assay

293T cells and JIP1−/− MEF cells transfected with appropriate plasmids were stimulated by MLK3 expression and glucose starvation, respectively, and were further grown for cell counting. Dead cells were stained with SYTOX Green (Invitrogen) in DMEM and visualized using a DE/Axiovert 200 inverted microscope (Carl Zeiss). Cells were counted in DIC and green fluorescence images to calculate cell death percentages in triplicate. Cell death assays were repeated at least four times.

## Results and Discussion

### JIP1-dependent JNK pathway was restored by replacing JIP1-JNK docking interaction with heterologous PDZ interaction domains

**Figure 1:**
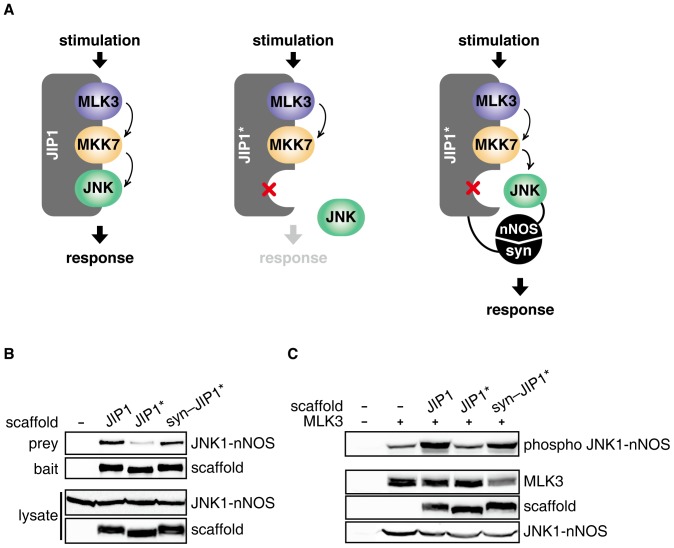
JNK MAP kinase pathway was restored by replacing JIP1-JNK docking interaction with PDZ interaction domains. A. The wiring strategy of the JNK MAP kinase pathway is illustrated. The diagram shows a conventional JIP1-dependent JNK MAP kinase pathway (left). A JIP1* scaffold containing mutations to disrupt docking interaction with JNK leads to release of JNK from the scaffold complex and, therefore, a decrease in the signaling response (middle). The functional scaffold complex is reassembled by re-recruiting missing JNK to the scaffold complex using fusions of syn-JIP1* and JNK1-nNOS, resulting in the restoration of JNK signaling (right). To recruit JNK to JIP1*, syn and nNOS PDZ domains were fused to JIP1* and JNK, respectively. B. JIP1, JIP1* or syn-JIP1* scaffold was co-expressed with Flag-JNK1-nNOS in 293T cells. Myc-tagged scaffold proteins were immunoprecipitated using an anti-myc antibody conjugated to agarose. The bound Flag-JNK1-nNOS was detected by immunoblotting. C. To verify that the alternatively assembled scaffold complex restored the signaling response, dual-phosphorylation of JNK1-nNOS in the whole cell lysates was detected by western blot analysis using an anti-phospho JNK antibody. Expression of HA-MLK3 was used to stimulate the JIP1-mediated JNK pathway in 293T cells [13]. Each experiment described here was performed in triplicate.

A major role of scaffold proteins in cell signaling is thought to be tethering of signaling components into an active complex via non-covalent interactions [3], [4], [24]. To investigate the assembly rules of JIP1 scaffold complex, we attempted to wire the JIP1-dependent JNK MAP kinase signaling pathway by replacing JIP1-JNK docking interaction using heterologous interaction modules (Figure 1A). A mutant of JIP1 (JIP1*), containing point mutations R156G and P157G in the JBD, was constructed to disrupt JIP1-JNK docking interaction [25]. The binding affinity of JIP1* for JNK was significantly decreased compared to wild-type JIP1 (Figure 1B). To recruit missing JNK to the scaffold complex, JIP1* and JNK were fused to PDZ domains from syntrophin (syn) and neuronal nitric oxide synthase (nNOS), respectively. When the fusions of syn-JIP1* and JNK1-nNOS were co-expressed in 293T cells, the interaction between JIP1* and JNK was successfully restored via PDZ interactions (Figure 1B). To determine whether the recruitment of JNK to JIP1* via a PDZ interaction module is sufficient for restoring signaling output, the dual-phosphorylation of JNK was monitored in cells expressing JNK1-nNOS along with JIP1, JIP1* or syn-JIP1*. Upon stimulation by MLK3 over-expression, which is known to trigger JIP1-dependent JNK signaling in 293T cells [13], [26]–[28], JIP1* led to a noticeable decrease in JNK phosphorylation, which was presumably due to the defective docking interaction. Surprisingly, syn-JIP1* expressed along with JNK1-nNOS was able to restore phosphorylation of JNK comparable to that by wild-type JIP1 (Figure 1C). Similar results were observed in COS7 cells, where the JIP1-JNK docking interaction was replaced by the identical set of PDZ domains (Figure S1 in File S1). Together, these data indicate that the JIP1-JNK docking interaction is highly modular and that the pathway connectivity of JNK signaling can be restored via alternative assembly of the JIP1 complex using heterologous protein interactions.

### JNK pathway was restored via alternative assembly of JIP1 complex using JBDs

**Figure 2:**
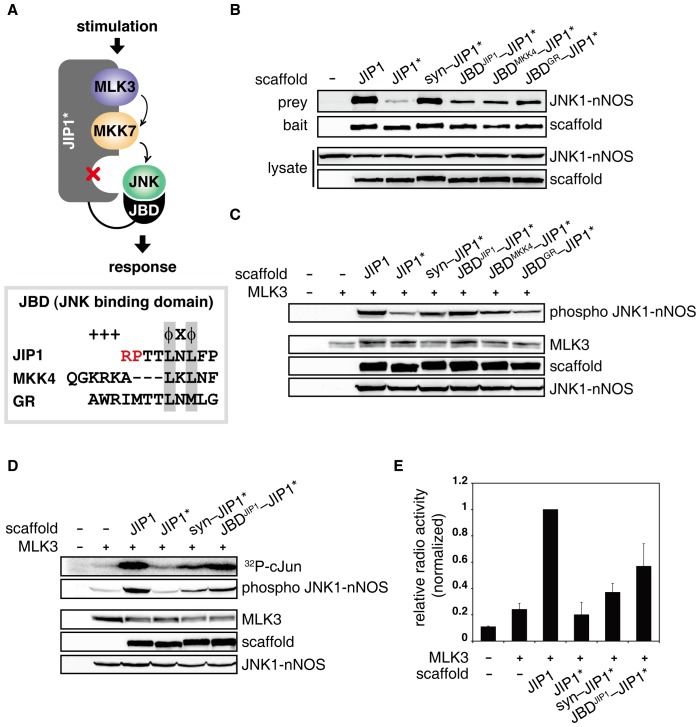
JNK MAP kinase pathway was restored by re-recruiting JNK using JBDs. A. The diagram illustrates the reassembly of JIP1 scaffold complex by re-recruiting the missing JNK using JBDs. Peptides encoding JBDs were fused to the N-terminus of JIP1* to recruit JNK to the scaffold complex. The box shows the alignment of the amino acid sequences of the following JBDs used in this experiment: mouse JIP1 (156–164), human MKK4 (38–48), and mouse GR (571–582). Basic (+) and hydrophobic (Φ) residues in the consensus sequence were indicated. Conserved hydrophobic residues among the sequences were boxed in gray. Arginine and proline residues mutated in JIP1* were indicated in red. B. Flag-JNK1-nNOS was co-expressed with JIP1 variants (JIP1, JIP1*, syn-JIP1*, JBD^JIP1^-JIP1*, JBD^MKK4^-JIP1* or JBD^GR^-JIP1*) in 293T cells. Interactions between JNK1-nNOS and JIP1 variants were analyzed as described in Figure 1B. C. Dual phosphorylation of Flag-JNK1-nNOS in the whole cell lysates was analyzed as described above to monitor the JNK signaling restored by reassembly of JIP1 complex via JBDs. Expression of HA-MLK3 was used to stimulate JIP1-mediated JNK pathway in 293T cells [13]. D and E. JNK signaling mediated by JIP1 scaffold reassembly was monitored by an in-gel kinase assay to measure the catalytic activity of activated JNK in the whole cell lysates. cJun protein was used as a substrate for JNK. Expressed proteins and phosphorylated JNK were examined by immunoblotting. Samples were separated in a gel containing GST-cJun, and catalytic activity of JNK was quantified by measuring the radioactivity incorporated in GST-cJun using a liquid scintillation counter. Each experiment was performed in triplicate and repeated at least three times.

The JNK-binding domain (JBD), which is conserved in many JNK-interacting proteins, provided an alternative tool for wiring the defective JIP1 assembly. To test whether JIP1 complex can be reassembled by protein interactions other than the PDZ domains, we attempted to re-recruit JNK to JIP* using JBDs from JIP1 (JBD^JIP1^), MKK4 (JBD^MKK4^) and GR (JBD^GR^) (Figure 2A). JBD-JIP1* fusions were expressed with JNK1-nNOS in 293T cells and tested for their ability to recruit the missing JNK to JIP1 complex. The immunoprecipitation results showed that the affinities of JBD^JIP1^-JIP1*, JBD^MKK4^-JIP1* and JBD^GR^-JIP1* for JNK1-nNOS were similar but not as strong as that of syn-JIP1* (Figure 2B). Although JBD^GR^-JIP1* did not restore JNK pathway, JBD^JIP1^-JIP1* and JBD^MKK4^-JIP1* were able to induce phosphorylation of JNK1-nNOS. Moreover, JBD^JIP1^-JIP1* triggered a higher level of JNK phosphorylation than syn-JIP1* did (Figure 2C, D). The results indicated that despite a weaker binding affinity than PDZ domains, JBD^JIP1^ is better optimized to activate the terminal MAP kinase, JNK. Similar results were observed when JNK2-nNOS was used instead of JNK1-nNOS (Figure S2 in File S1). A kinase assay was performed for JNK1 to test the ability of various recruiting modules to generate signaling. The results were consistent with the observations described above; JBD^JIP1^-JIP1* led to a higher level of JNK kinase activity than syn-JIP1* did (Figure 2D, E). To exclude the possibility that the remaining part of JBD in JIP1* might participate in, or interfere with, the recruitment and activation of JNK, a mutant of JIP1 in which the entire JBD was deleted (JIP1ΔJBD) was constructed, and its ability to restore signaling was compared with the JIP1* fusions. We observed that JIP1ΔJBD alone led to a decreased level of JNK phosphorylation comparable to JIP1*. Furthermore, syn-JIP1ΔJBD and JBD^JIP1^-JIP1ΔJBD exhibited similar effects on JNK phosphorylation, compared to syn-JIP1* and JBD^JIP1^-JIP1*, respectively (Figure S3 in File S1). These data indicate that the remaining part of JBD in JIP1* did not influence the rewiring by syn-JIP1* or JBD^JIP1^-JIP1*.

Together, these results suggested that the main function of the JIP scaffold is likely to recruit pathway components in close proximity via a modular assembly complex, and such modular interactions can also evolve to actively participate in catalysis. It is surprising that the JIP1-JNK docking interaction is highly modular, considering the fact that the JNK MAP kinase, which is the terminal component of the cascade, is the subject of complicated regulation mechanisms, including feedback control and crosstalk with other pathways [29].

### Reassembly of JIP1 scaffold complex via heterologous interaction domains restored cell death response

**Figure 3:**
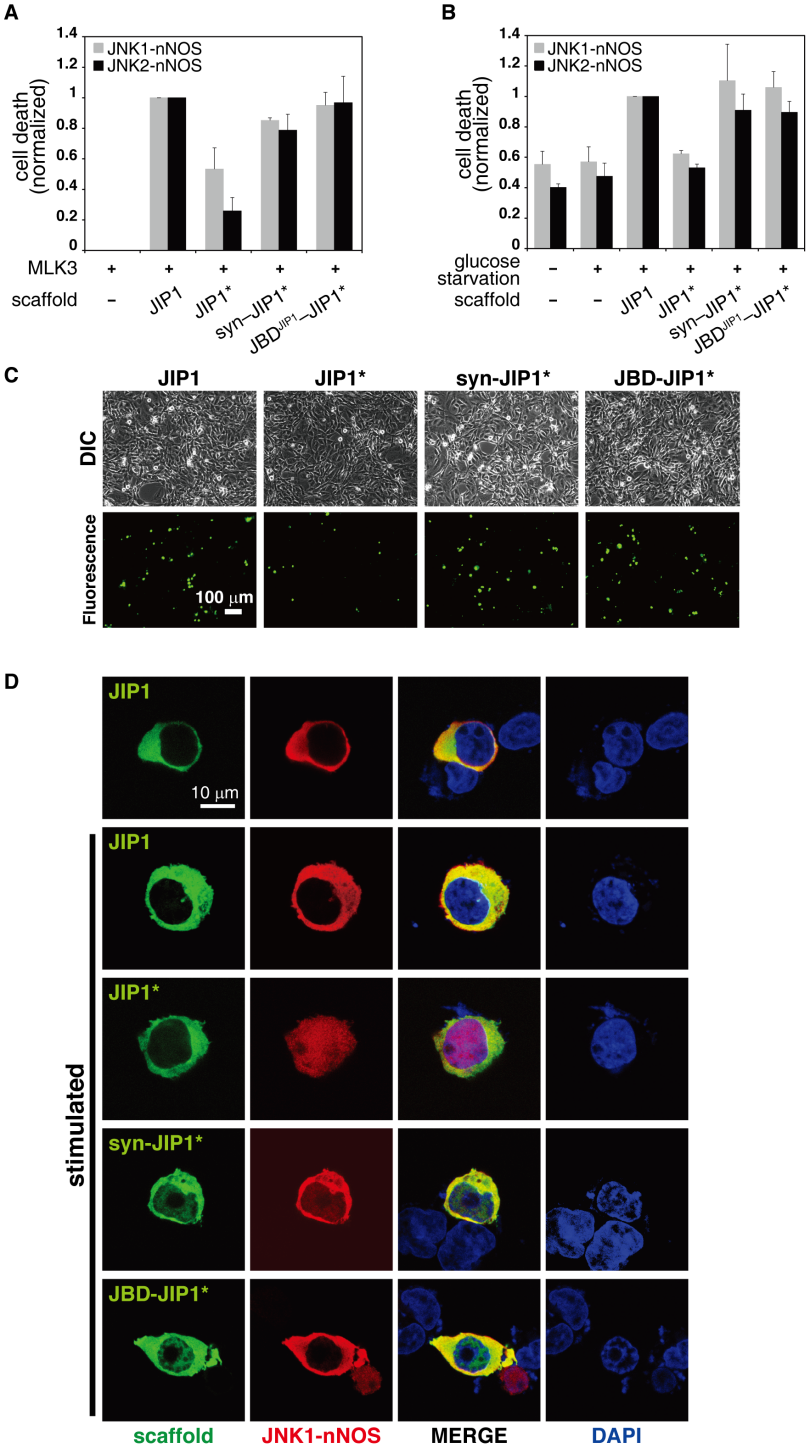
JNK signaling restored by alternative assembly of JIP complex was able to induce cell death and alter subcellular localization of JNK. Cell death responses were measured by counting cells stained with SYTOX Green. The relative ratios of cell death are plotted for JNK1-nNOS and JNK2-nNOS. The data in the bar graphs are the mean ±SD of triplicate experiments. A. Flag-JNK1-nNOS or Flag-JNK2-nNOS was co-expressed with JIP1 variants in 293T cells. Dead cells were counted after 24 hours of transfection. Expression of HA-MLK3 was used to stimulate JIP1-mediated JNK pathway in 293T cells [13]. B. Flag-JNK1-nNOS or Flag-JNK2-nNOS was co-expressed with variants of the JIP1 scaffold in JIP1−/− MEF cells. Cell death responses of MEF cells were examined after 20 hours of stimulation by glucose starvation. C. DIC and green fluorescence images were obtained for the MEF cells described above using a fluorescence microscope. A scale bar corresponding to 100 µm is indicated. D. Recruitment of JNK1 to JIP1 variants (JIP1, JIP1*, syn-JIP1* or JBD^JIP1^-JIP1*) via heterologous interaction modules was monitored in 293T cells. Cells were stimulated by expression of HA-MLK3 and were fixed with paraformaldehyde after 24 hours of transfection. JIP1 variants tagged with GFP were visualized by monitoring green fluorescence. Flag-tagged JNK1-nNOS was visualized using an anti-Flag antibody and TRITC-linked secondary antibody. Localization of JIP1 variants and JNK1-nNOS was examined from fluorescence images obtained using a confocal microscope. A scale bar corresponding to 10 µm is indicated. The images were obtained from more than 100 cells and all the cells examined exhibited similar patterns, of which representative images were shown here and in the Figure S6 in File S1.

Chronic activation of JNK signaling is known to induce apoptosis [10], [11], [27], and therefore, we attempted to test whether cell death response can be triggered by reassembly of JIP1 scaffold complex. When 293T cells expressing the appropriate scaffold and JNK fusions were stimulated, the presence of JIP1* resulted in a dramatic decrease in cell death response compared to JIP1 [28]. However, both syn-JIP1* and JBD^JIP1^-JIP1* were able to fully restore the cell death response with JNK1-nNOS or JNK2-NOS (Figure 3A). To rule out any effects from endogenous JIP1, we also examined cell death in JIP1−/− MEF cells stimulated by glucose starvation [30]. As JIP1 is known to be involved in JNK activation by glucose starvation, the cell death response could be triggered when a functional JIP complex was assembled in the MEF cells. Similar to the results from the experiments in 293T cells, syn-JIP1* and JBD^JIP1^-JIP1* resulted in the full restoration of cell death responses in JIP1−/− MEF cells (Figure 3B, C). Although syn-JIP1* was less efficient than JBD^JIP1^-JIP1* in JNK phosphorylation (Figure 2D), it generated similar levels of cell death response. These data demonstrated that JNK signaling by restored by reassembly of JIP1 scaffold complex is sufficient for causing physiological changes in cells.

### Subcellular localization of JNK MAP kinase was altered according to reassembly of JIP1 scaffold complex

To verify whether the subcellular localization of JNK1-nNOS was altered upon recruitment to a scaffold complex via heterologous interaction modules, we performed immunocytochemistry analysis for 293T cells (Figure 3D) and COS7 cells (Figure S5 in File S1) expressing JNK1-nNOS with or without various scaffold fusions (JIP1, JIP1*, Syn-JIP1*, or JBD^JIP1^-JIP1*). In the absence of JIP1 expression in unstimulated cells, most of JNK1 remained in both the cytoplasm and nucleus. Similar results were observed for JNK1-nNOS in both 293T cells and COS7 cells, indicating that the fusion with nNOS PDZ domain did not interfere with the subcellular localization of JNK1 (Figure S4 in File S1). When co-expressed with the wild-type JIP1 scaffold, JNK1-nNOS was sequestered from the nucleus regardless of stimulation, indicating that JIP1 caused JNK1-nNOS to be localized to the cytoplasm through the docking interaction between JIP1 and JNK1 (Figure 3D, JIP1 panel). It is not surprising to find that JNK1-nNOS was not clearly detectable in the nucleus upon stimulation, because only a trace amount in the cellular JNK pool is thought to be activated and shuttled to nucleus in a JIP1-dependent manner [10]. In contrast, co-expression of JIP1* did not alter the localization of JNK1-nNOS in the nucleus and cytoplasm, presumably due to the disruption of JNK1 recruitment by docking mutations in JIP1* (Figure 3D, JIP1* panel). However, co-expression of syn-JIP1* or JBD^JIP1^-JIP1* restored the sequestration of JNK1-nNOS in the cytoplasm to levels comparable to wild-type JIP1. This finding demonstrated that JNK1-nNOS can be re-recruited to the scaffold complex in cytoplasm via heterologous interaction modules (Figure 3D, bottom panels). In all cases, variants of JIP1 remained exclusively in the cytoplasm. Together, these data indicated that changes in the localization of JNK1-nNOS correlate well with the status of scaffold assembly by the JIP1 variants.

## Conclusions

In this study, we demonstrate that by using a JIP1 mutant defective in recruitment of JNK MAP kinase, a functional scaffold complex can be reassembled by recruiting the missing JNK via heterologous protein interactions, which suggests a modular assembly mechanism of JIP1 complex. The fact that heterologous interaction domains can functionally replace the JIP1-JNK docking interaction in the complex is surprising, in light of the many postulated regulatory mechanisms for JIP1 and JNK [11], [29]. The modular assembly of JIP1 scaffold complex demonstrated in this study is somewhat consistent with recent studies of Ste5 scaffold in yeast mating MAP kinase signaling, in which docking interactions of MAPKKK (Ste11) and MAPKK (Ste7) to Ste5 scaffold were functionally replaced, except for the terminal MAP kinase (Fus3) [9]. Together, our findings suggest that signaling pathways in mammals have evolved to accommodate demands for more complex physiological needs by allowing modularity in the JIP1-JNK docking interaction at the cascade's bottom tier. We also demonstrate that such an alternative assembly of JIP1 complex can generate signaling outputs and cause alterations in cellular physiology, including cell death. The modular and flexible nature of JIP1 scaffold assembly revealed in this study allows scaffolds to be used as a platform for the artificial manipulation of mammalian signaling.

## Supporting Information

File S1 **Figure S1, JNK pathway was rewired via alternative assembly of JIP1 scaffold complex in COS7 cells.** Flag-JNK1-nNOS was co-expressed with JIP1 variants (JIP1, JIP1*, syn-JIP1*, or JBD^JIP1^-JIP1*) in COS7 cells. HA-MLK3 was expressed to stimulate JIP1-mediated JNK pathway. Dual phosphorylation of JNK1-nNOS in the whole cell lysates was detected by immunoblotting using an anti-phospho JNK antibody after 24 hours of transfection. The experiment was performed in triplicate. Figure S2, JNK phosphorylation was rescued by the recruitment of JNK2 to JIP1* using PDZ domains or JBDs. Flag-JNK2-nNOS was co-expressed with JIP1 variants (JIP1, JIP1*, syn-JIP1*, JBD^JIP1^-JIP1*, JBD^MKK4^-JIP1* or JBD^GR^-JIP1*) in 293T cells. HA-MLK3 was expressed to stimulate JIP1-mediated JNK pathway. Expression of proteins and phosphorylation of Flag-JNK2-nNOS in the whole cell lysates were examined after 24 hours of transfection by immunoblotting. The experiment was performed in triplicate. Figure S3, The rest of JBD in JIP1* did not interfere with rewiring of JNK phosphorylation by syn-JIP1* or JBD^JIP1^-JIP1*. The entire JBD was deleted in JIP1 to create JIP1ΔJBD. Flag-JNK1-nNOS was expressed with JIP1 variants (JIP1, JIP1*, syn-JIP1*, JBD^JIP1^-JIP1*, syn-JIP1ΔJBD, JBD^JIP1^-JIP1ΔJBD or JIP1ΔJBD) in 293T cells. HA-MLK3 was expressed to stimulate JIP1-mediated JNK pathway. Expression of these proteins and phosphorylation of Flag-JNK1-nNOS were tested after 24 hours of transfection by immunoblotting. The experiment was performed in triplicate. **Figure S4, JNK1 and JNK1-nNOS showed similar cellular localization regardless of fused nNOS PDZ domain.** DsRed-JNK1 or dsRed-JNK1-nNOS was expressed in 293T cells and COS7 cells. Unstimulated cells were fixed with paraformaldehyde after 24 hours of transfection. After DAPI staining of nuclei, localization of expressed proteins was examined by a fluorescence microscopy. A scale bar corresponding to 10 µm is indicated. Each experiment was repeated at least three times. **Figure S5, JNK signaling wired by alternative assembly altered subcellular localization of JNK1-nNOS in COS7 cells.** The recruitment of JNK1 to JIP1 variants (JIP1, JIP1*, syn-JIP1* or JBD^JIP1^-JIP1*) via heterologous interaction modules was monitored in COS7 cells. Flag-JNK1-nNOS was co-expressed with JIP1 variants. Expression of HA-MLK3 was used to stimulate JIP1-mediated JNK pathway. Cells were fixed with paraformaldehyde after 24 hours of transfection. JIP1 variants tagged with GFP were visualized by monitoring green fluorescence. Flag-tagged JNK1-nNOS was visualized using anti-Flag antibody and TRITC-linked secondary antibody. The localization of JIP1 variants and JNK1-nNOS was examined from fluorescence images obtained using a confocal microscope. **Figure S6, Alternative assembly of JIP1 complex altered subcellular localization of JNK1 in 293T cells.** The recruitment of JNK1 to JIP1 variants, JIP1 and JIP1*, was monitored in 293T cells. Cells were fixed with paraformaldehyde after 24 hours of transfection. JIP1 variants tagged with GFP were visualized by monitoring green fluorescence. About 100 cells were monitored to determine the localization of proteins and the representative images are shown from a lower magnification of ×20 using dsRed-JNK1 (A) and from a higher magnification of ×40 using Flag-tagged JNK1 (B), which was visualized using anti-Flag antibody and TRITC-linked secondary antibody. The images of JIP1 variants and JNK1-nNOS were obtained using a fluorescence microscopy. A scale bar corresponding to 10 µm is indicated. **Table S7, List of plasmids used in this study.**
(DOCX)
