# Supplementary material for: Reassembly of JIP1 Scaffold Complex in JNK MAP Kinase Pathway Using Heterologous Protein Interactions
Source: PLoS One. 2014 May 9;9(5):e96797. doi: 10.1371/journal.pone.0096797 (PMC4016011; doi:10.1371/journal.pone.0096797)

**Supporting Information**

**Figures**


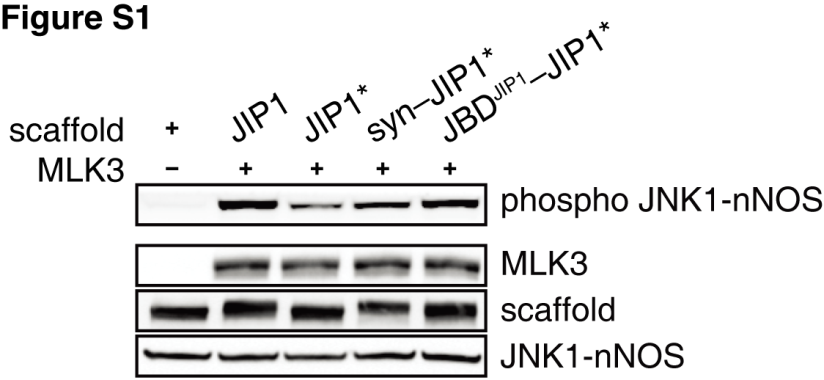


**Figure S1. JNK pathway was rewired via alternative assembly of JIP1 scaffold complex in COS7 cells.** Flag-JNK1-nNOS was co-expressed with JIP1 variants (JIP1, JIP1*, syn-JIP1*, or JBD^JIP1^-JIP1*) in COS7 cells. HA-MLK3 was expressed to stimulate JIP1-mediated JNK pathway. Dual phosphorylation of JNK1-nNOS in the whole cell lysates was detected by immunoblotting using an anti-phospho JNK antibody after 24 hours of transfection. The experiment was performed in triplicate.


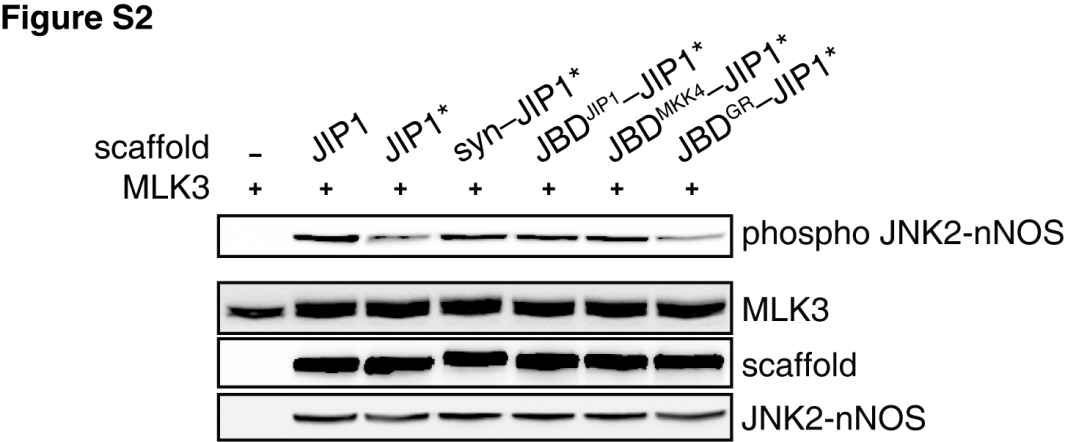


**Figure S2. JNK phosphorylation was rescued by the recruitment of JNK2 to JIP1* using PDZ domains or JBDs.** Flag-JNK2-nNOS was co-expressed with JIP1 variants (JIP1, JIP1*, syn-JIP1*, JBD^JIP1^-JIP1*, JBD^MKK4^-JIP1* or JBD^GR^-JIP1*) in 293T cells. HA-MLK3 was expressed to stimulate JIP1-mediated JNK pathway. Expression of proteins and phosphorylation of Flag-JNK2-nNOS in the whole cell lysates were examined after 24 hours of transfection by immunoblotting. The experiment was performed in triplicate.


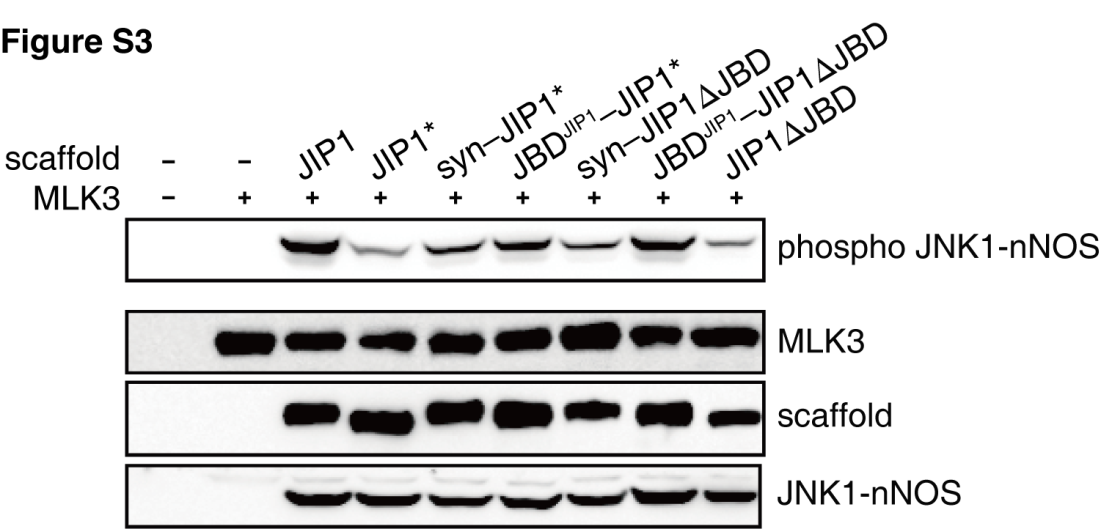


**Figure S3. The rest of JBD in JIP1* did not interfere with rewiring of JNK phosphorylation by syn-JIP1* or JBD^JIP1^-JIP1*.** The entire JBD was deleted in JIP1 to create JIP1ΔJBD. Flag-JNK1-nNOS was expressed with JIP1 variants (JIP1, JIP1*, syn-JIP1*, JBD^JIP1^-JIP1*, syn-JIP1ΔJBD, JBD^JIP1^-JIP1ΔJBD or JIP1ΔJBD) in 293T cells. HA-MLK3 was expressed to stimulate JIP1-mediated JNK pathway. Expression of these proteins and phosphorylation of Flag-JNK1-nNOS were tested after 24 hours of transfection by immunoblotting. The experiment was performed in triplicate.


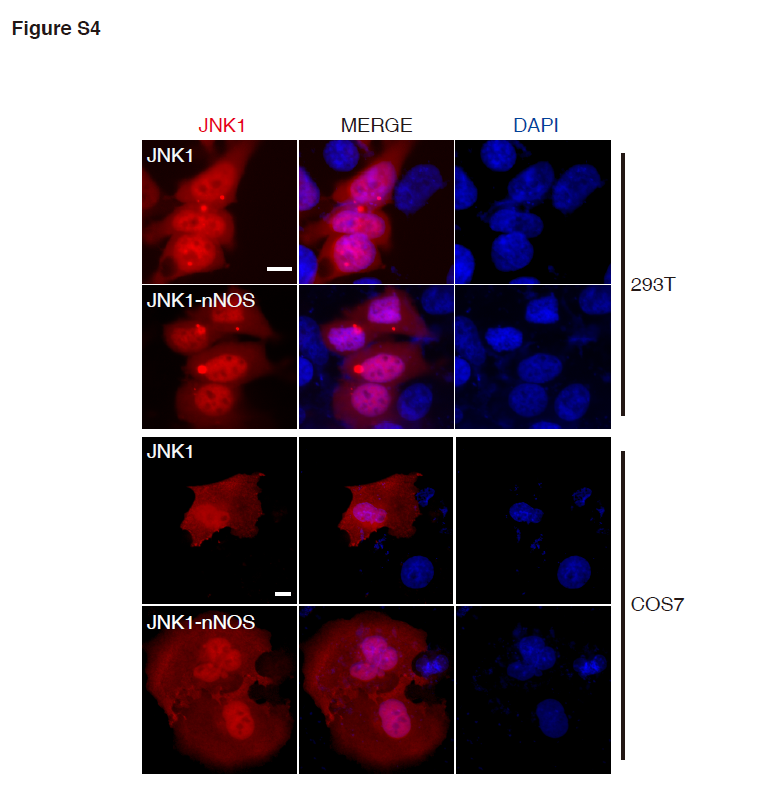


**Figure S4. JNK1 and JNK1-nNOS showed similar cellular localization regardless of fused nNOS PDZ domain.** DsRed-JNK1 or dsRed-JNK1-nNOS was expressed in 293T cells and COS7 cells. Unstimulated cells were fixed with paraformaldehyde after 24 hours of transfection. After DAPI staining of nuclei, localization of expressed proteins was examined by a fluorescence microscopy. A scale bar corresponding to 10 μm is indicated. Each experiment was repeated at least three times.


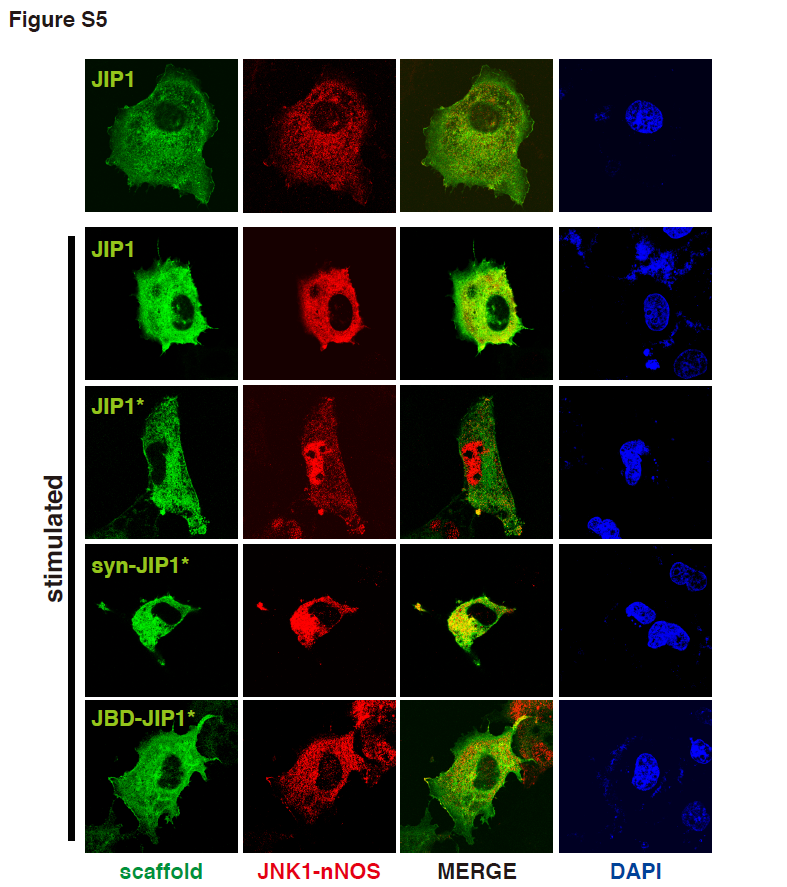


**Figure S5. JNK signaling wired by alternative assembly altered subcellular localization of JNK1-nNOS in COS7 cells.** The recruitment of JNK1 to JIP1 variants (JIP1, JIP1*, syn-JIP1* or JBD^JIP1^-JIP1*) via heterologous interaction modules was monitored in COS7 cells. Flag-JNK1-nNOS was co-expressed with JIP1 variants. Expression of HA-MLK3 was used to stimulate JIP1-mediated JNK pathway. Cells were fixed with paraformaldehyde after 24 hours of transfection. JIP1 variants tagged with GFP were visualized by monitoring green fluorescence. Flag-tagged JNK1-nNOS was visualized using anti-Flag antibody and TRITC-linked secondary antibody. The localization of JIP1 variants and JNK1-nNOS was examined from fluorescence images obtained using a confocal microscope.


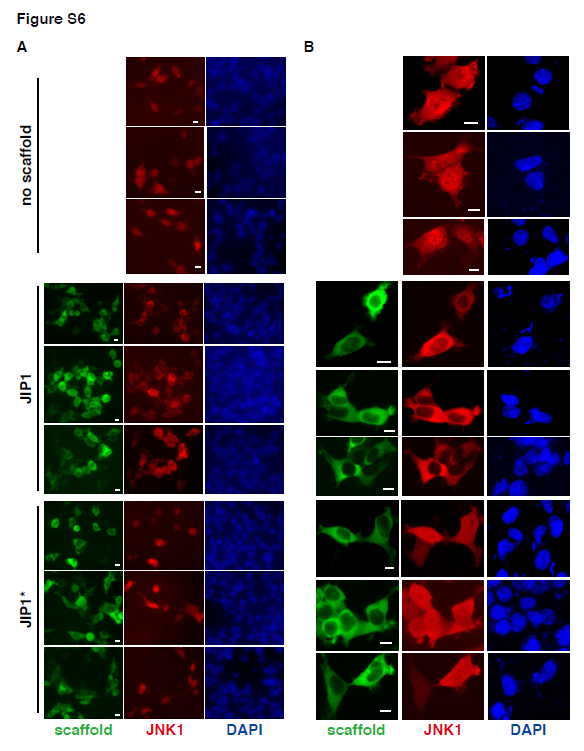


**Figure S6. Alternative assembly of JIP1 complex altered subcellular localization of JNK1 in 293T cells.** The recruitment of JNK1 to JIP1 variants, JIP1 and JIP1*, was monitored in 293T cells. Cells were fixed with paraformaldehyde after 24 hours of transfection. JIP1 variants tagged with GFP were visualized by monitoring green fluorescence. About 100 cells were monitored to determine the localization of proteins and the representative images are shown from a lower magnification of X20 using dsRed-JNK1 (A) and from a higher magnification of X40 using Flag-tagged JNK1 (B), which was visualized using anti-Flag antibody and TRITC-linked secondary antibody. The images of JIP1 variants and JNK1-nNOS were obtained using a fluorescence microscopy. A scale bar corresponding to 10 μm is indicated.

**Table S7. List of plasmids used in this study**


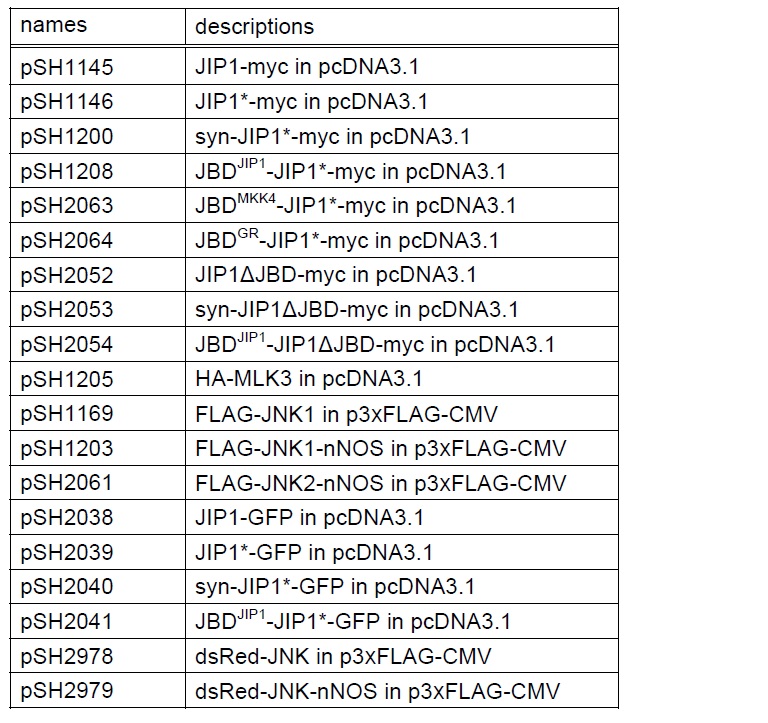

Supplement: File S1 — Figure S1, JNK pathway was rewired via alternative assembly of JIP1 scaffold complex in COS7 cells. Flag-JNK1-nNOS was co-expressed with JIP1 variants (JIP1, JIP1*, syn-JIP1*, or JBDJIP1-JIP1*) in COS7 cells. HA-MLK3 was expressed to stimulate JIP1-mediated JNK pathway. Dual phosphorylation of JNK1-nNOS in the whole cell lysates was detected by immunoblotting using an anti-phospho JNK antibody after 24 hours of transfection. The experiment was performed in triplicate. Figure S2, JNK phosphorylation was rescued by the recruitment of JNK2 to JIP1* using PDZ domains or JBDs. Flag-JNK2-nNOS was co-expressed with JIP1 variants (JIP1, JIP1*, syn-JIP1*, JBDJIP1-JIP1*, JBDMKK4-JIP1* or JBDGR-JIP1*) in 293T cells. HA-MLK3 was expressed to stimulate JIP1-mediated JNK pathway. Expression of proteins and phosphorylation of Flag-JNK2-nNOS in the whole cell lysates were examined after 24 hours of transfection by immunoblotting. The experiment was performed in triplicate. Figure S3, The rest of JBD in JIP1* did not interfere with rewiring of JNK phosphorylation by syn-JIP1* or JBDJIP1-JIP1*. The entire JBD was deleted in JIP1 to create JIP1ΔJBD. Flag-JNK1-nNOS was expressed with JIP1 variants (JIP1, JIP1*, syn-JIP1*, JBDJIP1-JIP1*, syn-JIP1ΔJBD, JBDJIP1-JIP1ΔJBD or JIP1ΔJBD) in 293T cells. HA-MLK3 was expressed to stimulate JIP1-mediated JNK pathway. Expression of these proteins and phosphorylation of Flag-JNK1-nNOS were tested after 24 hours of transfection by immunoblotting. The experiment was performed in triplicate. Figure S4, JNK1 and JNK1-nNOS showed similar cellular localization regardless of fused nNOS PDZ domain. DsRed-JNK1 or dsRed-JNK1-nNOS was expressed in 293T cells and COS7 cells. Unstimulated cells were fixed with paraformaldehyde after 24 hours of transfection. After DAPI staining of nuclei, localization of expressed proteins was examined by a fluorescence microscopy. A scale bar corresponding to 10 µm is indicated. Each experiment was repeated at least three times [file pone.0096797.s001.docx]
